# Assessment of Multi-Mycotoxin Exposure in Southern Italy by Urinary Multi-Biomarker Determination

**DOI:** 10.3390/toxins6020523

**Published:** 2014-01-28

**Authors:** Michele Solfrizzo, Lucia Gambacorta, Angelo Visconti

**Affiliations:** Institute of Sciences of Food Production (ISPA), National Research Council (CNR), Bari 70126, Italy; E-Mails: lucia.gambacorta@ispa.cnr.it (L.G.); angelo.visconti@ispa.cnr.it (A.V.)

**Keywords:** mycotoxins, biomarker, urine, UPLC-MS/MS, immunoaffinity cleanup, exposure

## Abstract

Human exposure assessment to deoxynivalenol (DON), aflatoxin B1 (AFB1), fumonisin B1 (FB1), zearalenone (ZEA) and ochratoxin A (OTA) can be performed by measuring their urinary biomarkers. Suitable biomarkers of exposure for these mycotoxins are DON + de-epoxydeoxynivalenol (DOM-1), aflatoxin M1 (AFM1), FB_1_, ZEA + α-zearalenol (α-ZOL) + β-zearalenol (β-ZOL) and OTA, respectively. An UPLC-MS/MS multi-biomarker method was used to detect and measure incidence and levels of these biomarkers in urine samples of 52 volunteers resident in Apulia region in Southern Italy. The presence of ZEA + ZOLs, OTA, DON, FB1 and AFM1 were detected in 100%, 100%, 96%, 56% and 6%, of samples, respectively. All samples contained biomarkers of two or more mycotoxins. The mean concentrations of biomarkers ranged from 0.055 ng/mL (FB1) to 11.89 ng/mL (DON). Urinary biomarker concentrations were used to estimate human exposure to multiple mycotoxin. For OTA and DON, 94% and 40% of volunteers, respectively exceeded the tolerable daily intake (TDI) for these mycotoxins. The estimated human exposure to FB1 and ZEA was largely below the TDI for these mycotoxins for all volunteers.

## 1. Introduction

Aflatoxins, deoxynivalenol (DON), zearalenone (ZEA), fumonisins and ochratoxin A (OTA) are recognized as the principal mycotoxins occurring in agricultural products and their levels in food commodities are constantly inspected worldwide. Humans can be daily exposed to mixtures of these mycotoxins through consumption of foods contaminated with several mycotoxins or consumption of different foods contaminated by a single mycotoxin. Data on the co-occurrence of the principal mycotoxins in foods and beverages are increasing due to the availability and use of modern and sensitive LC-MS/MS methodologies suitable for simultaneous determination of mycotoxins and other fungal metabolites [1,2]. In a recent survey on 265 samples of cereal-based products commercialized in Spain, Italy, Marocco and Tunisia, 14% of the analyzed samples were contaminated with at least two mycotoxins and 18% of the analyzed samples were contaminated by more than two mycotoxins simultaneously [3]. The co-occurrence of DON, ZEA and nivalenol (NIV) in winter wheat produced in Sweden has been recently reported [4]. The presence of mixtures of aflatoxin B1 (AFB_1_), ZEA and OTA was reported in samples of breakfast cereals commercialized in Spain [5]. The majority of food commodities consumed in Cameroon were found contaminated with mixtures of mycotoxins, 21% contained DON, ZEA and fumonisin B1 (FB1), 11% DON, AFB1, FB1 and ZEA [6]. Recently, the co-occurrence of DON, FB1, fumonisin B2 (FB2), fumonisin B3 (FB3) and ZEA in good and moldy maize and DON, FB1, FB2, ZEA and OTA in samples of maize based foods was reported in the former Transkei region of South Africa [7,8].

Exposure to mycotoxins can also originate from the ingestion of their masked forms (mycotoxins covalently or non-covalently bound to matrix component) that are digested in the gastrointestinal tract and released into the parent mycotoxins that become bioavailable. The occurrence of masked DON and ZEA, either acetylated, conjugated with glucose or sulfate, has been reported in various cereals and food samples [9]. The occurrence of masked fumonisins in processed food has also been reported but the nature of the masking mechanism has not been fully clarified [10]. The formation of β-glucosides of (4R)- and (4S)-5-hydroxy-OTA in germinating cereals and vegetables spiked with OTA has been demonstrated but the occurrence of these compounds in naturally contaminated food has not been reported [11]. The degree of human bioavailability of mycotoxins derived from their masked forms is not known and could vary between individuals depending of the intestinal microbiota composition. 

The assessment of human exposure to mycotoxins is usually performed by means of chemical analysis of foods and beverages and results are correlated with the mean intake of analyzed foods/beverages. The heterogeneous distribution of mycotoxins in food samples can affect the accuracy of results obtained with this approach. Duplicate diet studies could avoid sampling issues but requires suitable analytical methods for single or multi-mycotoxin determination and considerable commitment from the participants. 

The measurement of specific urinary mycotoxin biomarkers is a valid alternative to measure exposure to mycotoxins providing that the excretion of biomarkers correlate well with mycotoxin intake. Suitable urinary biomarkers for AFB1, FB1, ZEA, DON and OTA are aflatoxin M1 (AFM1), FB1, ZEA + α-zearalenol (α-ZOL) + β-zearalenol (β-ZOL), DON + de-epoxydeoxynivalenol (DOM-1) and OTA, respectively. These biomarkers are excreted as free and conjugated forms therefore urine samples are usually digested with β-glucuronidase/sulfatase in order to deconjugate the conjugated forms and increase the concentration and detectability of free analytes. Human pilot studies and epidemiological studies based on biomarker approach have been performed for DON, AFB1, OTA and FB1. These studies were conducted by using analytical methods tailored for determination of biomarker(s) of a single mycotoxin [12,13,14,15]. LC-MS/MS is the ideal approach for simultaneous determination of analytes and its use for multi-biomarker determination in human and animal urine is recently increased [8,16]. *In vivo* experiments demonstrated a good correlation between the amount of mixtures of DON, OTA, ZEA, FB1 and AFB1 administered to piglets and the amount of relevant biomarkers excreted in 24 h post dose urine. Linear dose-response correlation coefficients ranged between 0.68 and 0.78 for the tested couples of mycotoxin/biomarker. Mean percentages of dietary mycotoxins excreted as biomarkers in 24 h post dose urine were 36.8% for ZEA, 28.5% for DON, 2.6% for FB1, 2.6% for OTA and 2.5% for AFB1 [17]. In this paper, we report the results on the occurrence of biomarkers to DON, OTA, ZEA, FB1 and AFB1 in urine samples of 52 volunteers resident in Apulia a region of Southern Italy. 

## 2. Results and Discussion

The urinary concentrations of mycotoxin biomarkers could be very low for several reasons: (a) the levels of mycotoxins in foods and beverages can be very low especially for AFB1, OTA and ZEA; (b) the gastrointestinal absorption can be low as demonstrated for FB1, c) the serum half-life can be very high as demonstrated for OTA. The purification protocol used in our study for simultaneous determination of urinary DON, DOM-1, AFM1, FB1, ZEA, α-ZOL, β-ZOL and OTA was successfully validated in a mini comparison study involving other laboratories that used either another multi-biomarker method or single-biomarker methods for determination of DON and FB1 [18]. To improve the sensitivity of the method we used an UPLC system coupled with a powerful and sensitive mass spectrometer (API 5000 MS/MS system with ESI interface). It was necessary to optimize the chromatographic conditions for optimal biomarker separation on Acquity BEH phenyl column as well as the MS/MS conditions. The optimized MS/MS conditions for each biomarker are reported in Table 1. 

The main differences with the MS/MS parameters previously optimized on a QTrap system [19] were: (a) the increase in number of daughter ions from 3 to 4 for FB1, α-ZOL and β-ZOL; (b) minor modification in the clustering potential, entrance potential, collision energy and collision cell exit potential (Table 1). The use of the Acquity BEH phenyl column and the development of a new linear gradient composition of the mobile phase permitted to reduce the run time from 46 min to 15 min. However, each sample extract was analyzed twice in positive and negative mode. AFM1, FB1 and OTA were detected and measured in positive mode whereas DON, DOM-1, ZEA, α-ZOL and β-ZOL were detected and measured in negative mode. The use of an UPLC system permitted to use a 150 mm × 2.1 mm column with a 1.7 µm particles size of stationary phase that produced sharp peak thus increasing peak high with consequent reduction of the limit of detection (LOD) and quantification (LOQ). The use of a powerful mass spectrometer (API 5000 MS/MS system) produced a further increase in method sensitivity. In particular, a marked increase in sensitivity (up to 114 times) was obtained, in descending order, for ZEA, β-ZOL, α-ZOL, FB1, AFM1 and OTA with the new UPLC-MS/MS system as compared to the previous one (Table 2). No increase of sensitivity was obtained for DON whereas for DOM-1 an increase of LOQ value was observed probably due to ion suppression effect. 

**Table 1:** MS/MS conditions for detection of target analytes by MRM method.

Analyte	Precursor ion	Q1 (m/z)	Q3 (m/z)	DP (V)	EP (V)	CE (V)	CXP (V)
DON	[DON+CH_3_COO]^−^	355.0	295.2	−50	−10	−15	−20
			265.0 ^a^			−22	
			59.0 ^a^			−35	
DOM-1	[DOM-1+CH_3_COO]^−^	339.5	279.0	−50	−10	−12	−20
			249.2	−50		−18	
			59.0 ^a^	−55		−35	
AFM1	[AFM1+H]^+^	329.5	273.3 ^a^	80	10	36.5	20
			229.0 ^a^			56	
FB1	[FB1+H]^+^	722.4	370.6 ^a^	50	10	55	10
			352.6 ^a^			59	
			334.7 ^a^			65	
			316.6 ^a^			66	
α-ZOL	[α-ZOL-H]^−^	319.1	188.2 ^a^	−100	−10	−38	−10
			174.1 ^a^			−36	
			160.1 ^a^			−43	
			130.0 ^a^			−48	
β-ZOL	[β-ZOL-H]^−^	319.1	188.2 ^a^	−100	−10	−38	−10
			174.1 ^a^			−36	
			160.1 ^a^			−43	
			130.0 ^a^			−48	
ZEA	[ZEA-H]^−^	317.2	273.3	−100	−10	−29	−10
			175.0			−35	
			131.0 ^a^			−40	
OTA	[OTA+H]^+^	404.2	358.5	90	10	30	10
			257.3			38	
			239.2 ^a^			47	

Notes: ^a^ Transitions used for quantitation; *Q1*: first quadrupole; *Q3*: third quadrupole; *DP*: declustering potential; *EP*: entrance potential; *CE*. collision energy; *CXP*. collision cell exit potential

**Table 2:** LOQ values for DON, AFM1, FB1, β-ZOL, α-ZOL, ZEA and OTA in human urine samples obtained with two different LC-MS/MS systems: LC-QTrap MS/MS system and UPLC-API 5000 MS/MS system.

Biomarker	Apparatus 1: LC-QTrap MS/MS LOQ (ng/mL)	Apparatus 2: UPLC-API 5000 MS/MS LOQ (ng/mL)
ZEA	0.8	0.007
β-ZOL	4.4	0.054
α-ZOL	1.6	0.030
FB1	0.1	0.010
AFM1	0.1	0.020
OTA	0.02	0.006
DON	1.5	1.5
DOM-1	1.5	9.9

**Figure 1:**
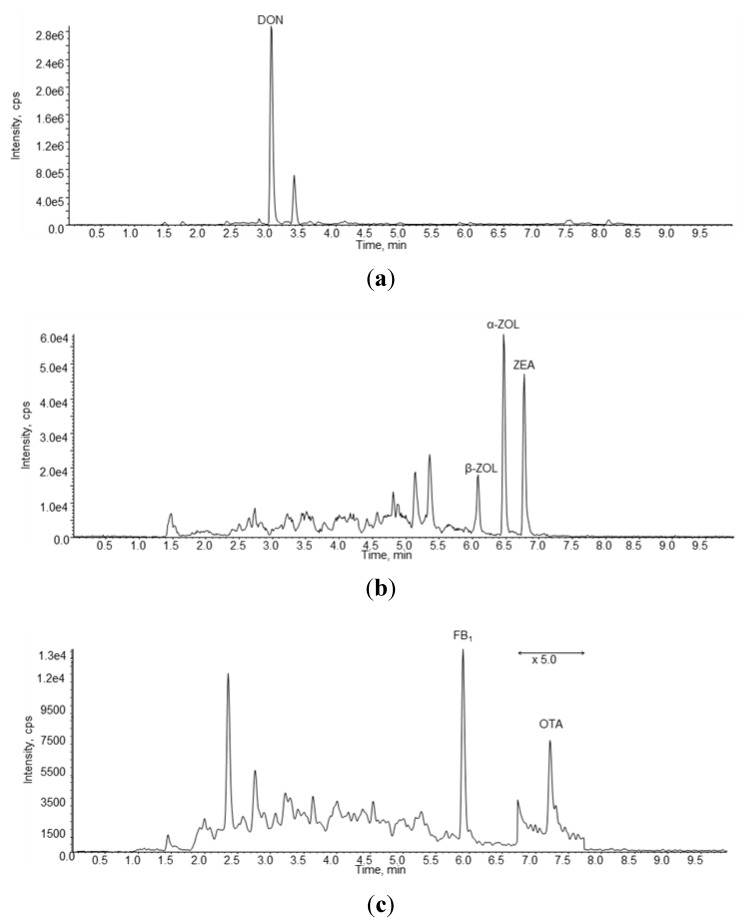
LC-MS/MS chromatograms obtained in negative ion mode (**a**,**b**) and in positive ion mode (**c**) of a naturally urine sample (#4) containing 11.33 ng/mL of DON, 0.108 ng/mL of β-ZOL, 0.123 ng/mL of α-ZOL, 0.082 ng/mL of ZEA, 0.26 ng/mL of FB1 and 0.06 ng/mL of OTA.

In Figure 1 are reported three chromatograms of a naturally contaminated urine sample containing DON, ZEA, α-ZOL, β-ZOL, FB1 and OTA. 

A summary of the results of the urine samples collected in this study are reported in Table 3. All urine samples contained ZEA, α-ZOL and OTA whereas β-ZOL was found in 98% of samples. DON, FB1 and AFM1 were found in 96%, 56% and 6% of urine samples, respectively. The highest mean biomarker concentration was found for DON (11.89 ng/mL) followed by OTA (0.144 ng/mL), β-ZOL (0.090 ng/mL), α-ZOL (0.077 ng/mL), AFM1 (0.068 ng/mL), ZEA (0.057 ng/mL) and FB1(0.055 ng/mL). The highest individual biomarker concentration was measured for DON (67.36 ng/mL) followed by OTA (2.129 ng/mL), FB1 (0.352 ng/mL), α-ZOL (0.176 ng/mL), β-ZOL (0.135 ng/mL), AFM1 (0.146 ng/mL) and ZEA (0.120 ng/mL). From this Table it is evident that urinary concentrations of DON are much higher to those of the other biomarkers. Moreover, the highest inhomogeneous distributions of concentrations was observed for OTA as demonstrated by the value of relative standard deviation (RSD) of the mean (217%) followed by FB1 (133%), AFM1 (99%), DON (84%), ZEA (40%), α-ZOL (35%) and β-ZOL (16%). 

**Table 3:** Results of mycotoxin biomarkers in human urine samples collected in Southern Italy and estimated values of PDI for each mycotoxin

Biomarkers	DON	β-ZOL	α-ZOL	ZEA	FB1	OTA	AFM1
N. positive (%)	50 (96)	51 (98)	52 (100)	52 (100)	29 (56)	52 (100)	3 (6)
Mean, ng/mL	11.89	0.090	0.077	0.057	0.055	0.144	0.068
SD, ng/mL	10.05	0.014	0.027	0.023	0.073	0.312	0.067
Median, ng/mL	10.32	0.088	0.074	0.056	0.029	0.061	0.10
Max, ng/mL	67.36	0.135	0.176	0.120	0.352	2.129	0.146
**Mycotoxins**	**DON**	**ZEA**			**FB1**	**OTA**	**AFB1**
Mean PDI^a^, µg/kg body weight	1.03	0.015			0.053	0.139	0.068
Max PDI^a^, µg/kg body weight	5.90	0.029			0.338	2.047	0.142
% of PDI^a^ values exceeding the TDI	40	0			0	94	0
Mean PDI^b^, µg/kg body weight	0.59	--^c^			0.274	--^c^	--^c^
Max PDI^b^, µg/kg body weight	3.37	--^c^			1.759	--^c^	--^c^
% of PDI^b^ values exceeding the TDI	6	--^c^			0	--^c^	--^c^
TDI^d^, µg/kg body weight	1.0	0.2			2.0	0.017	--^e^

Notes: ^a^ calculated based on piglet excretion data; ^b^ calculated based on human excretion data (50% for DON, 0.5% for FB1) reported in Shephard *et al.* [8]; ^c^ not estimated due to unavailability of human excretion rate; ^d^ TDI values are reported in [19] and references therein; ^e^ there is no TDI value for AFB1 because it is a carcinogenic mycotoxin.

The results of the co-occurrence of multiple biomarkers in the tested urine samples are reported in Table 4. The majority of urine samples (52%) contained biomarkers of DON, ZEA, FB1 and OTA whereas 38% of samples contained biomarkers of DON, ZEA and OTA. The co-occurrence of biomarkers of all mycotoxins was found in two urine samples. Moreover, no individual was found unexposed or exposed to a single mycotoxin since all investigated urine samples contained biomarkers of at least two mycotoxins. No important differences were observed for the results obtained for male and female individuals with the exception that the two urine samples containing biomarkers of all mycotoxins were from females. Mixtures of biomarkers of DON, ZEA, FB1 and OTA were found in human urine samples collected in the former Transkei, South Africa [8]. Co-occurrence of biomarkers of 2 to 5 mycotoxins was reported in human urine samples collected in Cameroon [20,21].

**Table 4:** Incidence of individuals exposed to mixtures of mycotoxins for a total of 52 volunteers (26 males and 26 females).

Multiple mycotoxins exposure	n. positive samples	% of positive samples
DON, ZEA, FB1, OTA, AFB1	2	4
DON, ZEA, FB1, OTA	27	52
DON, ZEA, OTA	20	38
DON, ZEA, OTA, AFB1	1	2
ZEA, OTA	2	4
TOTAL	52	100

This is the first report on the simultaneous detection of biomarkers of the 5 principal mycotoxins in Italy. The simultaneous presence of DON and OTA in human urine in Italy was previously reported [19] whereas the presence of ZEA, α-ZOL, β-ZOL, FB1 and AFM1 is reported herein for the first time. The presence of DON and OTA in almost all urinary samples is not surprising because these mycotoxins are usually found in cereals and derived products, staple foods of Italian people [22,23]. Interestingly, 56% of urine samples contained FB1, a mycotoxin mainly found in maize and deriving products. Although these products are not staple foods in Italy they are widely consumed as chips, polenta, popcorn, beer, cornflakes, snacks, muesli and mixed cereals [22,24]. The consumption of these products could explain the high percentage of urine samples containing FB1. The low concentrations of biomarkers of ZEA in all tested urine samples is also a new and interesting information and demonstrate that all the 52 volunteers that participated in our study were exposed to low levels of ZEA. On the other hand the results of a large survey conducted in Europe showed that only 32% of 5018 samples of cereals and derived products, the main source for ZEA exposure, were found contaminated with this mycotoxin [22]. A possible explanation of this apparent paradox could be that the high sensitivity of the multi biomarker method used in the present study can detect urinary biomarker deriving from the consumption of foods contaminated with very low levels of ZEA that could not be detected with conventional analytical methods. The presence of AFM1 in only 3 urine samples demonstrates that human exposure to AFB1 is quite limited in this area of Southern Italy. In fact ground nuts and tree nuts, the main source of human AFB1 exposure in Italy, are occasionally consumed in this Country. The absence of AFM1 in almost all urine samples suggests that maize and derived products consumed in Southern Italy are probably not contaminated with AFB1 although it is well known that maize is a major source of AFB1 in some countries. The absence of AFM1 in human urine and AFB1 in maize based food was also reported in South Africa [8]. 

The urinary biomarker concentrations measured in this study were used to estimate the probable daily intake (PDI) of each mycotoxin by each volunteer according to Equation (1).

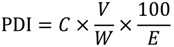
(1)


PDIprobable daily intake of mycotoxin (µg/kg body weight);*C*human urinary biomarker concentration (µg/L);*V*mean 24 h human urine volume (1.5 L);*W*mean human body weight (60 kg);*E*mean urinary excretion rate of mycotoxin in 24 h post dose in piglets (36.8% for ZEA, 27.9% for DON, 2.6% for FB1, 2.6% for OTA and 2.5% for AFM1 [17]).

Based on mean urinary concentration of each mycotoxin biomarker measured in the 52 human urine samples and the mean urinary excretion rate in piglets for each mycotoxin, the estimated daily mean intake of the 5 investigated mycotoxins were calculated and are reported in Table 3. Due to the unavailability of human excretion rate for all the 5 mycotoxins considered in this study we used the 24 h excretion rate measured in piglets [17] to estimate the PDI in human. However, since sufficient data of human excretion rate of DON and FB1 have been reported [8], additional values of PDI, calculated with human data, were added in Table 3. In this table, the values of max PDI for each mycotoxin and the percentage of individuals that exceeded the tolerable daily intake (TDI) for each mycotoxin are also reported. The estimated mean values of PDI were below or equal to the TDI or provisional maximum TDI (PMTDI) for DON, FB1 and ZEA. Individual analysis of PDI values obtained for DON revealed that 40% of volunteers exceeded the value of TDI of 1 µg/kg body weight established for this mycotoxin with a maximum value of PDI of 5.90 µg/kg body weight. Previous studies conducted in UK, France and Sweden using the urinary DON concentration to estimate DON exposure reported mean values of PDI of 0.12–0.73, 0.61 and 0.16 µg/kg body weight, respectively [25,26,27,28,29,30]. Human exposure to DON in our study seems to be higher to that estimated in UK since both mean values of PDI and % of individuals that exceed TDI are higher as compared to UK where some 5% of the adult population may exceed the TDI for DON intake [25]. The mean PDI values of DON derived from food analyses in UK (0.14–0.23 µg/kg body weight) seems to be slightly lower as compared to PDI values estimated from urinary DON in that Country [25,26,27,28,31]. The mean PDI values of DON derived from food analysis in Europe and reported by SCOOP report [22] and FAO/WHO [32] were 0.34 and 1.4 µg/kg body weight, respectively. The results of our study obtained with the biomarker approach fall within this interval and is more close to the PDI reported by FAO/WHO [32]. A lower mean value of PDI (0.59 µg/kg body weight) was obtained by using the human excretion rate of 50% reported by Shephard *et al.* [8] as well as the percentage of individuals (6%) that exceeded the value of TDI for this mycotoxin.

The mean value of PDI for FB1 calculated in our study with biomarker approach (0.053 µg/kg body weight) is far below the TDI established for this mycotoxin (2 µg/kg body weight) and all individual values are below the TDI for this mycotoxin with a maximum value of PDI of 0.338 µg/kg body weight (Table 3). These results can only be compared with results obtained in Guatemala, South Africa and Mexico because urinary FB1 in European population has not been performed yet. The mean values of PDI estimated with biomarker approach in Guatemala, Mexico and South Africa were 0.45, 0.37 and 0.22 µg/kg body weight, respectively [15,33,34]. Comparison with these data shows that in our study human exposure to FB1 is about 10 times lower to those estimated in Guatemala, Mexico and South Africa. The mean PDI values of FB1 obtained in our study with biomarker approach (0.053 µg/kg body weight) is quite similar to the PDI of 0.056 µg/kg body weight estimated with diet approach by Brera *et al.* in Italy [24]. The estimated mean PDI value reported by FAO/WHO [32] for Europe with the diet approach was 0.2 µg/kg body weight which is 3.8 times higher than the intake estimated in our study. A much higher mean value of PDI (0.274 µg/kg body weight) was obtained by using the human excretion rate of 0.5% reported by Shephard *et al.*, [8]. However even with this estimate all individuals resulted below the value of TDI for this mycotoxin. 

The mean value of PDI of ZEA estimated with biomarker approach in our study (0.015 µg/kg body weight) is about ten times below the TDI established for this mycotoxin (0.2 µg/kg body weight). The maximum estimated value of PDI is 0.029 µg/kg body weight (Table 3). In Italy, the mean value of PDI estimated with the diet approach was 0.0008 µg/kg body weight [22] whereas the PDI reported by EFSA for Europe was 0.03–0.06 µg/kg body weight [35]. The mean value of PDI estimated in our study is higher than the Italian value reported in the SCOOP report and lower than the lower limit of the range reported by EFSA [35]. 

The occurrence of ZEA biomarkers in human urine was recently reported in USA and Cameroon. Mixtures of ZEA, α-ZOL and β-ZOL were detected and measured in 55% of urine samples collected from girls resident in New Jersey with mean concentrations ranging from 0.35 ng/mL (β-ZOL) to 1.82 ng/mL (ZEA) [36]. These concentrations are 4–32 times higher the mean concentrations found in our study for these ZEA biomarkers which means that in New Jersey human exposure to ZEA is higher as compared to Italy. Mixtures of ZEA, α-ZOL and ZEA-glucuronide were found in a low percentage (5%) of human urine samples collected in Cameroon with a total mean concentration of 0.74 ng/mL [20].

As reported in Table 3 the mean value of PDI of OTA estimated with biomarker approach in our study (0.139 µg/kg body weight) is about 8 times higher than the TDI (0.017 µg/kg body weight) established for this mycotoxin [37]. The maximum value of PDI estimated in our study (2.047 µg/kg body weight) is 147 times higher than the TDI and 94% of individuals participating in our study exceeded the value of TDI (Table 3). Several studies reported the occurrence of OTA in human urine with a high percentage of positive samples but the relevant values of PDI from these results were not calculated nor reported. We used the human urinary concentrations of OTA reported in literature to estimate values of PDI of OTA by using the Equation (1) reported above. Urinary concentrations of OTA reported by Fazekas *et al.* [38] and Duarte *et al.* [14,39] produced values of PDI equal or below the TDI, whereas from the results of Pena *et al.* [40], Manique *et al.* [41], Coronel *et al.* [42], Gilbert *et al.* [43] and Domijan *et al.* [44] the estimated values of PDI were higher the TDI. Our result of estimated mean PDI, though higher than the TDI, is lower than the mean PDI values estimated from the urinary concentrations reported by Domijan *et al.* in Croatia [44] and Coronel *et al.* in Spain [42]. When compared to the PDI values estimated with urinary concentrations reported by Fazekas *et al.* [38], Duarte *et al.* [14,39], Pena *et al.* [40], Manique *et al.* [41] and Gilbert *et al.* [43] our estimated PDI values were higher. 

In Europe, the mean values of PDI of OTA estimated with the diet approach range from 0.0011 to 0.024 µg/kg body weight [23]. The mean value of PDI estimated with our biomarker study (0.139 µg/kg body weight) is 5.8–127 times higher than the European PDI values estimated with diet approach. All together, these data clearly show that the estimated human exposure to OTA is higher when using the biomarker approach as compared to the diet approach. Possible explanations of these differences could be: a) the urinary excretion rate of OTA in piglets, used in this study to estimate PDI values, is completely different from that in human, b) the food diet approach did not cover all sources of OTA exposure due to the occurrence of this mycotoxin in a very high number of different foods and beverages. It is well known that OTA is the mycotoxin more widespread in several different types of foods and beverages [23,32].

The results shown in Table 3 indicate that AFM1 was detected and measured in only 3 urine samples (6% of total samples) which confirm that human exposure to AFB1 was sporadic. The relevant mean value of PDI of AFB1, calculated for the 3 positive subjects, is 0.068 µg/kg body weight. As reported above, there is no TDI value for AFB1 because this is a carcinogenic mycotoxin. The incidence of positive samples and mean urinary AFM1 concentration measured in positive samples in our study are comparable to those reported by Hatem *et al.* from Marasmus, Egypt [45]. Polychronaki *et al.* reported, for Guinea, a mean urinary concentration of AFM1 in positive samples similar to those found in our study but a higher percentage of positive samples (64%) [13]. The mean value of PDI of AFB1 estimated with biomarker approach for the three volunteers participating in our study (0.068 µg/kg body weight) is largely higher than the PDI of aflatoxin intake measured with the diet approach in Europe (0.00016–0.00055 µg/kg body weight) [23].

## 3. Experimental Section

### 3.1. Chemicals and Reagents

Standard solutions were purchased from Romer Labs Diagnostic (Tulln, Austria). In particular, solutions of DON (100 µg/mL), DOM-1 (50 µg/mL), AFM1 (0.5 µg/mL), ZEA (100 µg/mL), α-ZOL (10 µg/mL), β-ZOL (10 µg/mL) and OTA (10 µg/mL) were prepared in acetonitrile (ACN) whereas FB1 solution (50 µg/mL) were prepared in acetonitrile-water (50:50). β-glucuronidase/sulfatase type H-2 from *Helix pomatia* (specific activity 130,200 units/mL β-glucuronidase, 709 units/mL sulfatase). Chromatography-grade methanol (MeOH) and glacial acetic acid were obtained from Carlo Erba (Milan, Italy). Ultrapure water was produced by use of a Milli-Q system (Millipore, Bedford, MA, USA). Myco6in1^®^ immunoaffinity columns were purchased from Vicam L.P (Watertown, MA, USA). OASIS^®^ HLB columns, 60 mg, 3 mL were purchased from Waters (Milford, MA, USA) and regenerated cellulose filters (0.45 µm) were purchased from Sartorius Stedim Biotech (Goettingen, Germany).

### 3.2. Equipment and Conditions

LC-MS/MS analyses were performed on a triple quadrupole API 5000 system (Applied Biosystems, Foster City, CA, USA), equipped with a ESI interface and an Acquity UPLC system comprising a binary pump and a microautosampler from Waters (Milford, MA, USA). The analytical column was an Acquity UPLC BEH phenyl column (2.1 mm × 150 mm, 1.7 µm particles; Waters). The column oven was set at 40 °C. The flow rate of the mobile phase was 250 µL/min and the injection volume was 10 µL. For multi-biomarker separation a multiple linear binary linear gradient of acidic MeOH (containing 0.5% acetic acid) in water (containing 0.5% acetic acid) was developed and used as mobile phase as follows: from 20% to 80% MeOH in 5 min, then maintained at 80% MeOH for 5 min, then brought to 20% MeOH in 0.5 min and left to equilibrate for 4.5 min before the next run. For LC-MS/MS analyses, the ESI interface was used in positive ion mode for AFM1, FB1 and OTA and in negative ion mode for DON, DOM-1, ZEA, α-ZOL and β-ZOL. The mass spectrometer operated in MRM (multiple reaction monitoring) mode and the optimized MS/MS conditions for each analyte are listed in Table 1. In particular, 4 transitions were monitored for confirmation of FB1, α-ZOL and β-ZOL whereas the sum of the 4 transitions were used for quantification; for DON 3 transitions for confirmation and the sum of 2 transition for quantification; for DOM-1, ZEA and OTA 3 transitions for confirmation and 1 transition for quantification; for AFM1 2 transitions for confirmation and the sum of 2 transition for quantification. Interface conditions were: TEM, 450 °C; CUR, nitrogen, 20 psi; GS1, air, 60 psi; GS2, air, 40 psi; ionspray voltage +5500 V or −4500 V. The signal of each compound was preliminary optimized with each proposed ionization condition. The tuning procedure included the optimization of source parameters during infusion of 1 µg/mL standard solution (0.1 µg/mL for AFM1) of the individual toxins in MeOH-water (20:80) containing 0.5% acetic acid at 10 µL/min, using an Harvard 11 plus infusion pump, into the UPLC mobile phase (50/50 water/methanol at 250 µL/min) by means of a minimum dead volume T-piece connected after the analytical column.

### 3.3. Participants and Urine Collection

For the evaluation of human exposure to DON, AFB1, FB1, ZEA and OTA 100 individuals residing in 10 municipalities of Puglia region (Southern Italy) were invited to participate in the urine sampling. Each individual was asked to provide a first morning urine sample and to fill out a questionnaire concerning age, gender and health status. The majority of individuals gave oral informed consent and donated a sample of first morning urine and the filled in questionnaire. Fifty-two individuals (participation rate 52%), 26 males and 26 females, (mean age 41 years, range 3–85 years) were recruited from municipalities of Bari, Triggiano, Mola di Bari, Monopoli, Adelfia, Conversano, Polignano a Mare, Bitonto, Martina Franca and Statte. The remaining 48 individuals failed to donate their morning urine samples. All participants collected their urine samples on 26 April 2011. Urine samples were stored at −18 °C until analysis for identification and determination of DON, DOM-1, AFM1, FB1, ZEA, α-ZOL, β-ZOL and OTA. Eleven volunteers declared to have health problems, in particular three individuals with hypertension, three with allergies, three with diabetes, one with hyperthyroidism and one at risk of thrombosis.

### 3.4. Calibration Curves

A mixed standard solution with a final concentration of 150 ng/mL DON, 20 ng/mL of DOM-1, 1.8 ng/mL AFM1, 28 ng/mL FB1, 35 ng/mL β-ZOL, 23 ng/mL α-ZOL, 12 ng/mL ZEA and 2 ng/mL OTA was prepared by mixing appropriate volumes of commercially available standard solutions and appropriate volume of ACN. Five standard calibration solutions covering appropriate range of analyte concentrations were prepared by portioning adequate volumes of mixed standard solution that were dried and reconstituted with 200 µL of initial LC-MS/MS mobile phase. In particular, the 5 standard calibration solutions were prepared by drying 25, 125, 250, 375 and 1000 µL of mixed standard solution that were reconstituted with 200 µL of initial LC-MS/MS mobile phase. Matrix-matched calibration solutions were prepared in 5 purified urinary extracts. In particular, urine from 6 individuals were pooled and mixed then 5 aliquots of 6 mL each were purified according to the protocol reported above. The 5 eluates collected from OASIS^®^ HLB and Myco6in1^®^ columns were spiked with 5 aliquots of the mixed standard solution (25, 125, 250, 375 and 1,000 µL), dried and reconstituted with 200 µL of initial LC-MS/MS mobile phase. The ranges of mycotoxin concentrations in the calibration solutions were, therefore: 18.75–750.00 ng/mL for DON, 2.50–100.00 ng/mL for DOM-1, 0.22–8.76 ng/mL for AFM1, 3.50–140.00 ng/mL for FB1, 4.38–175.00 ng/mL for β-ZOL, 2.88–115.00 ng/mL for α-ZOL, 1.50–60.00 ng/mL for ZEA and 0.25–10.00 ng/mL for OTA.

### 3.5. Analysis of Urinary Biomarkers

Urine samples were hydrolyzed with β-glucuronidase/sulfatase enzyme to hydrolyse glucuronide and/or sulfate conjugates of DON, DOM-1, ZEA, α-ZOL, β-ZOL and then purified with a multi-antibody and OASIS^®^ HLB columns according to the protocol reported elsewhere [19]. In brief, a 6 mL urine sample was hydrolyzed at 37 °C for 18 h with 300 µL of β-glucuronidase/sulfatase type H-2 from *Helix pomatia*. Hydrolyzed sample was diluted with 6 mL of water and purified on a Myco6in1^®^ and OASIS^®^ HLB columns connected in tandem. The OASIS^®^ HLB column was previously conditioned by passing 2 mL MeOH and 2 mL ultrapure water. After sample application and elution, the two columns were separated, the Myco6in1^®^ was washed with water (4 mL) and eluted with methanol (3 mL) and water (2 mL) that were collected in a vial. The OASIS^®^ HLB column was washed with methanol/water (20:80, 1 mL) and DON that had passed through the Myco6in1^®^ and retained on the OASIS^®^ HLB was eluted with methanol/water (40:60, 1 mL). The separate eluates from the two columns were combined, dried down and reconstituted in methanol/water (20:80, 200 µL) with 0.5% acetic acid. Purified extract was filtered through a regenerated cellulose filter and a volume of 10 µL (equivalent to 0.3 mL urine) was analyzed by UPLC-MS/MS.

## 4. Conclusions

The improved UPLC-MS/MS method for simultaneous determination of urinary biomarker for DON, FB1, OTA, AFB1 and ZEA was suitable to detect and accurately measure the low mycotoxin biomarker concentrations naturally occurring in the human urine samples collected in this study.

A multiple mycotoxin exposure was found for all tested volunteers participating in the study. 

This is the first report on the occurrence of urinary AFM1, FB1, ZEA and ZOLs in Italy. 

The estimated PDI values of OTA largely exceeded the TDI value for this mycotoxin in 94% of volunteers.

The mean estimated PDI of DON is similar to the TDI value for this mycotoxin but in 40% of volunteers it exceeded the value of TDI.

The values of PDI estimated herein with urinary biomarker approach matched quite well with the intake estimated with the diet approach reported in the literature for DON, FB1 and ZEA whereas for OTA and AFB1 the intake estimated with the biomarker approach was much higher than the intake estimated with the diet approach reported in the literature. 
